# Supplementary material for: An analytical theory of balanced cellular growth
Source: Nat Commun. 2020 Mar 6;11:1226. doi: 10.1038/s41467-020-14751-w (PMC7060212; doi:10.1038/s41467-020-14751-w)
Supplement: Supplementary file 1 — Supplementary Information [file 41467_2020_14751_MOESM1_ESM.pdf]

Supplementary Information:

An analytical theory of balanced cellular growth

Hugo Dourado & Martin J. Lercher

Contents

Supplementary Notes **2**

Supplementary Note 1: Definition of relative fitness 2

Supplementary Note 2: An outline of possible extensions of GBA 3

Supplementary Note 3: Growth Control Analysis (GCA) 5

Supplementary Note 4: Choice of basis, relationship between density and dependence constraints 7

Supplementary Figures **8**

Supplementary Tables **12**

Supplementary References **14**

Supplementary Notes

Supplementary Note 1: Definition of relative fitness

In a situation where competition among cells is solely through differential intrinsic growth rates, absolute fitness is equal to growth rate: In a population of cells growing exponentially with growth rate μ, the selection coefficient for a variant with growth rate $\mu + \delta\mu$ is simply $\delta\mu$[1]. Population genetics models almost always employ relative fitness[2], which we here define as a relative growth rate:

$$f \equiv \frac{\mu + \delta\mu}{\mu} = 1 + \frac{\delta\mu}{\mu} \quad .$$

Thus, to quantify the effect on relative fitness of a small change of some parameter x by δx, we use

$$\frac{\delta f}{\delta x} = \frac{1}{\mu}\frac{\delta\mu}{\delta x} \quad .$$

Note that population genetics models are frequently defined in terms of discrete generations. With generation time $T_{\mathrm{gen}} = \ln 2/\mu$, the selection coefficient of the variant *per generation* is then[3]

$$s_{\mathrm{T}} = (f-1)\ln 2 = \frac{\delta\mu}{\mu}\ln 2 \quad .$$

Supplementary Note 2: An outline of possible extensions of GBA

In our development of GBA, we make several simplifying assumptions. Here, we outline some possible generalizations.

All proteins contribute to growth by acting as catalysts or transporters. This assumption can simply be removed by adding a sector of non-growth related proteins[4,5] with concentration Q to the r.h.s. of equation (2).

Proteins are not used as reactants. To use protein j as a reactant in reaction j', it will need an extra row in A, and its concentration p_j has to enter the concentration vector $\mathbf{y} \equiv [P, p_j, \mathbf{a}]^T$ and the kinetic function $k_{j'}(p_j, \mathbf{a})$. This does not affect equation (4). However, if p_j appears on the right hand side of equation (5), this equation will have to be solved for p_j before it is possible to proceed to a generalization of the growth equation.

All catalysts are proteins. We can add different catalytic RNA species as cellular components. Additionally, we may introduce reactions that combine proteins and RNA into molecular machines such as the ribosome.

A 1-to-1 correspondence between proteins and reactions. Spontaneous reactions that proceed without a catalyst have to be included in the active stoichiometric matrix A (so that A^{-1} accounts for their dilution). They will need a kinetic function that relates their flux to the substrate concentrations (e.g., through mass action kinetics). However, they will not contribute to the protein sum (equation (2)) and hence will not directly contribute to the growth equation (6). Because in this case the flux cannot be adjusted by varying the concentration of a catalyst, only concentration vectors are feasible for which the flux through this reaction is identical when calculated based on mass conservation (through A^{-1}) and on kinetics. This will reduce the dimensionality of the solution space.

In the case of isoenzymes, where both protein j and protein j' catalyze the same reaction, the optimal solution will always use the one with the more favourable kinetics at the given reactant concentrations (e.g., protein j if $k_j(\mathbf{a}) > k_{j'}(\mathbf{a}) > 0$).

For protein complexes, where proteins j and j' have to bind to each other before they can act as a catalyst, we can either ignore the individual proteins and include the protein complex as a cellular component in the model, or add a reaction that describes the complex formation.

Finally, if one protein (or protein complex) catalyzes reactions j and j', the substrates (and possibly products) of reaction j' will enter the kinetic function $k_j(\mathbf{a})$. The fluxes through both reactions are proportional to the protein concentration p. Hence, $p = v_j/k_j(\mathbf{a}) = v_{j'}/k_{j'}(\mathbf{a})$, providing an additional constraint for the fluxes $v_j, v_{j'}$. As the fluxes are unique given the concentration vector $\mathbf{y} = [P, \mathbf{a}]^T$, again not all concentration vectors $\mathbf{y}$ will be compatible with this condition, reducing the dimensionality of the solution space of balanced growth.

Optimizing only growth. An extension of GBA can be formulated for non-growing cells (or cellular subsystems) that are instead optimized for the production of specific molecules, as is the case for many cell types in multicellular organisms. The dilution term $\mu\mathbf{y}$ in equation (1) would be replaced by a vector $\mathbf{d}(\mathbf{y})$ that quantifies the degradation of proteins and other molecules (with entries $d_i = z_i y_i$ and constant degradation rate z_i); an additional "output vector" $\mathbf{o}$ would represent the desired cellular production with rate v_o:

$$A\mathbf{v} = v_o\mathbf{o} + \mathbf{d}(\mathbf{y}) \quad .$$

The kinetics are still a function of **y**, and we can proceed with the analysis following the same steps as for equation (1) to calculate **v**, **p**, and v_o.

Supplementary Note 3: Growth Control Analysis (GCA)

We briefly explore the connection between GBA and some central concepts of *metabolic control analysis* (MCA)[6]. The results below that involve elasticities and control coefficients largely restate previous insights[7,8] in the framework of GBA. First, we rephrase the balance equation in terms of control theory.

We define the (scaled) *growth control coefficients* (GCC) as the *total* relative change in the growth rate due to a small change in the concentration x_i, *accounting for the density constraint.* The growth rate change is caused by two effects: the net fitness benefit of increasing x_i without considering the density constraint, captured by the marginal net benefits η_i; and the fitness cost of reducing the cellular density ρ available for all other concentrations, captured by $-\kappa_i\eta_\rho$. The GCC is then simply the sum of these two,

$$\Gamma_i^\mu = \eta_i - \kappa_i\eta_\rho \quad . \tag{1}$$

From the balance equation, we have $\Gamma_i^\mu = 0$ at optimal growth, so in real systems Γ_i^μ could be used as an objective measure of how "non-optimal" concentration x_i is. A related definition of growth control coefficients has been introduced before in the context of noise propagation in a model of gene expression and cellular growth[9].

We now examine the the relationship of the variables defined in GBA to the coefficients considered in MCA. The *elasticity coefficient* ε_α^j is defined in MCA as the change in the reaction rate j when varying the substrate concentration a_α while keeping the enzyme (catalyzing protein) concentration fixed[6]. The (scaled) elasticity coefficient is thus directly related to the marginal kinetic benefit u_α^j,

$$\varepsilon_\alpha^j \equiv \frac{1}{v_j}\left(\frac{\partial v_j}{\partial a_\alpha}\right)_{p_j=const} = \frac{1}{p_j k_j} p_j \frac{\partial k_j}{\partial a_\alpha} = \frac{1}{k_j}\frac{\partial k_j}{\partial a_\alpha} = \frac{u_\alpha^j}{\phi_j} \quad .$$

Control coefficients have been defined in MCA as the change in a *response variable* z due to a change in a *state variable* x_i, where each $z = z(\mathbf{x}, \pi)$ is a function of the state variables $\mathbf{x}$ and the *system parameters* π[6]. In the GBA framework, the growth rate μ, the fluxes $\mathbf{v}$, the protein concentrations $\mathbf{p}$, and the dependent concentrations $\mathbf{c}$ are all functions of the concentrations $\mathbf{x} = [P, \mathbf{b}]^T$, the active matrix A, and the kinetic parameters in $\mathbf{k}$. Thus, $\mu, \mathbf{v}, \mathbf{p}$, and $\mathbf{c}$ can be seen as response variables, while the concentrations x_i are state variables. In contrast to MCA, the GBA framework provides explicit functions for all response variables, and thus control coefficients can be calculated easily. The control of μ by the concentrations x_i is given by the growth control coefficient Γ_i^μ in the supplementary equation (1), while the control of dependent concentrations c_γ is directly determined by the dependence matrix D.

We next examine the control of fluxes v_j and protein concentrations p_j. The (scaled) *flux control coefficient* (FCC) $\Gamma_i^{v_j}$ is the relative change in v_j due to a small change in x_i (at fixed concentrations $x_{i'}$ for $i' \neq i$),

$$\Gamma_i^{v_j} \equiv \frac{1}{v_j}\frac{\partial v_j}{\partial x_i} \quad .$$

Using Theorem 5, we can calculate $\partial v_j / \partial x_i$, giving

$$\begin{aligned}\Gamma_i^{v_j} &\equiv \frac{1}{v_j}\frac{\partial \mu}{\partial x_i}\frac{v_j}{\mu} + \frac{\mu}{v_j}B_{ji}^{-1} = \frac{1}{\mu}\frac{\partial \mu}{\partial x_i} + \frac{\mu}{v_j}B_{ji}^{-1} \\ &= \Gamma_i^\mu + \frac{q_i^j}{\phi_j} \quad .\end{aligned}$$

At optimal growth, $\Gamma_i^{\mu} = 0$, so

$$\Gamma_i^{v_j} = \frac{q_i^j}{\phi_j} \quad .$$

Thus, at optimal growth, the flux control coefficient is simply the marginal production cost incurred via reaction j, divided by the proteome fraction of the catalyzing protein.

The (scaled) *protein control coefficient* (PCC) $\Gamma_i^{p_j}$ is the change in the proteome fraction of protein j, p_j/P, due to a small change in the concentration x_i (at fixed concentrations $x_{i'}$ for $i' \neq i$),

$$\Gamma_i^{p_j} \equiv \frac{1}{P}\frac{\partial p_j}{\partial x_i} \quad .$$

From the kinetic constraint (12),

$$\Gamma_i^{p_j} = \frac{1}{P}\frac{\partial}{\partial x_i}\left(\frac{v_j}{k_j}\right) = \frac{1}{P}\left(\frac{1}{k_j}\frac{\partial v_j}{\partial x_i} - \frac{v_j}{k_j^2}\frac{\partial k_j}{\partial x_i} + \sum_\gamma D_{\gamma i}\frac{v_j}{k_j^2}\frac{\partial k_j}{\partial c_\gamma}\right)$$
$$= \frac{1}{Pk_j}\frac{\partial v_j}{\partial x_i} - u_i^j - \sum_\gamma D_{\gamma i} u_\gamma^j \quad .$$

Again calculating $\partial v_j/\partial x_i$ using Theorem 5, we obtain

$$\Gamma_i^{p_j} = \frac{1}{Pk_j}\left(\frac{\partial \mu}{\partial x_i}\frac{v_j}{\mu} + \mu B_{ji}^{-1}\right) - u_i^j - \sum_\gamma D_{\gamma i}u_\gamma^j = \phi_j \Gamma_i^{\mu} + q_i^j - u_i^j - \sum_\gamma D_{\gamma i} u_\gamma^j$$
$$= \phi_j \Gamma_i^{\mu} - \eta_i^j \quad ,$$

where we defined $\eta_i^j \equiv -q_i^j + u_i^j + \sum_\gamma D_{\gamma i} u_\gamma^j$ as the contribution of reaction (or protein) j to the marginal net benefit η_i. Summing over j, we obtain

$$\sum_j \Gamma_i^{p_j} = \frac{\Gamma_i^{\mu}}{P}\sum_j p_j - \sum_j \eta_i^j$$
$$= \Gamma_i^{\mu} - \eta_i - \kappa_i \eta_\rho \quad . \tag{2}$$

Without a density constraint, $\eta_\rho = 0$, and

$$\sum_j \Gamma_i^{p_j} = 0 \quad . \tag{3}$$

Supplementary equations (2) and (3) can be seen as *summation theorems* that relate the GCC Γ_i^{μ} with the control coefficients of MCA, in a similar fashion as in Ref.[9].

At optimal growth,

$$\Gamma_i^{p_j} = -\eta_i^j \quad ,$$

and

$$\sum_j \Gamma_i^{p_j} = -\eta_i \quad .$$

Typically, reactants participate in only a small fraction of reactions, so for most combinations i, j, we have $u_i^j = 0$ and $D_{\gamma i} u_\gamma^j = 0$; the PCC at optimal growth is then just the marginal production cost,

$$\Gamma_i^{p_j} = q_i^j \quad .$$

Supplementary Note 4: Choice of basis, relationship between density and dependence constraints

Not every reactant can be considered dependent: a reactant for which the corresponding row in the active matrix A is linearly independent of all other rows will always be in the basis (equivalently, a reactant that has zero entries in all vectors in a basis for the left null space of A cannot be a dependent reactant).

For some models, it is possible that there is one or more choices of basis such that its corresponding dependence matrix has for some $i \in \{P, \beta\}$

$$\sum_{\gamma} D_{\gamma i} = -1 \quad .$$

In these cases, any marginal change in the mass concentration of component i will cause the exact opposite change in the total mass concentration of its dependent reactants γ. When this is combined with the density constraint as defined in equation (9), these changes in concentrations result in a perfect cancellation in the density utilized by i and its dependent reactants, and thus a zero net change in density for any change in the concentration i (i.e., $\kappa_i = 0$, Def. 4). For this reason, the marginal net benefit of i is simply $\eta_i = 0$ (Theorem 10).

Such a perfect cancellation is highly unlikely if we use a more realistic description of the density constraint, where different cellular components i have different specific density utilizations σ_i; e.g., if we assume that the density constraint limits the total volume occupied by cellular components, then σ_i gives the volume per mass of component i. In this case, the density constraint (14) (equation 9) is replaced by a volume density constraint, i.e., a constraint on the volume of cellular dry mass per volume of cell water, ν:

$$\nu \geq \sigma_{\mathrm{P}} P + \sum_{\alpha} \sigma_{\alpha} a_{\alpha} \quad ,$$

where σ_{P} is the specific density of proteins (almost constant for different proteins[10]) and σ_{α} is the specific density of reactant α, which depends on chemical properties such as hydrophobicity and charge[11].

Supplementary Figures

a

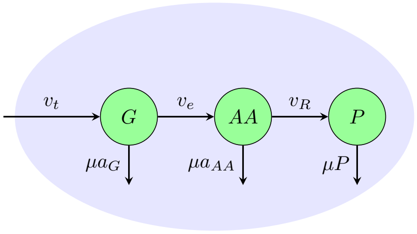

$$A = B = \begin{array}{c} \begin{matrix} t & e & R \end{matrix} \\ \begin{bmatrix} 1 & -1 & 0 \\ 0 & 1 & -1 \\ 0 & 0 & 1 \end{bmatrix} \end{array} \begin{matrix} G \\ AA \\ P \end{matrix}$$

$$A^{-1} = \begin{array}{c} \begin{matrix} G & AA & P \end{matrix} \\ \begin{bmatrix} 1 & 1 & 1 \\ 0 & 1 & 1 \\ 0 & 0 & 1 \end{bmatrix} \end{array} \begin{matrix} t \\ e \\ R \end{matrix}$$

Component	Marginal net benefits	Density factors
G	$\eta_G = \frac{1}{P}\left(\frac{p_e}{k_e}\frac{\partial k_e}{\partial a_G} - \frac{\mu}{k_t}\right)$	$\kappa_G = 1$
AA	$\eta_{AA} = \frac{1}{P}\left(\frac{p_R}{k_R}\frac{\partial k_R}{\partial a_G} - \frac{\mu}{k_t} - \frac{\mu}{k_e}\right)$	$\kappa_{AA} = 1$
P	$\eta_P = \frac{1}{P}\left(1 - \frac{\mu}{k_t} - \frac{\mu}{k_e} - \frac{\mu}{k_R}\right)$	$\kappa_P = 1$

---

b

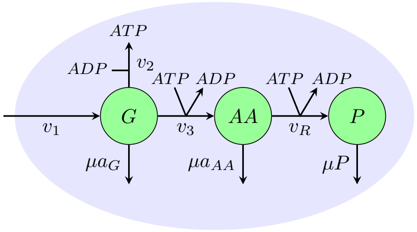

$$A = \begin{array}{c} \begin{matrix} 1 & 2 & 3 & R \end{matrix} \\ \begin{bmatrix} 1 & -0.5 & -0.5 & 0 \\ 0 & -0.5 & 0.5 & 0.5 \\ 0 & 1 & -0.5 & -0.5 \\ 0 & 0 & 0.5 & -0.5 \\ 0 & 0 & 0 & 0.5 \end{bmatrix} \end{array} \begin{matrix} G \\ ADP \\ ATP \\ AA \\ P \end{matrix} ,$$

$$B = \begin{array}{c} \begin{matrix} 1 & 2 & 3 & R \end{matrix} \\ \begin{bmatrix} 1 & -0.5 & -0.5 & 0 \\ 0 & 1 & -0.5 & -0.5 \\ 0 & 0 & 0.5 & -0.5 \\ 0 & 0 & 0 & 0.5 \end{bmatrix} \end{array} \begin{matrix} G \\ ATP \\ AA \\ P \end{matrix}$$

$$C = \begin{array}{c} \begin{matrix} 1 & 2 & 3 & R \end{matrix} \\ \begin{bmatrix} 0 & -0.5 & 0.5 & 0.5 \end{bmatrix} \end{array} \; ADP$$

$$B^{-1} = \begin{array}{c} \begin{matrix} G & ATP & AA & P \end{matrix} \\ \begin{bmatrix} 1 & 0.5 & 1.5 & 2 \\ 0 & 1 & 1 & 2 \\ 0 & 0 & 2 & 2 \\ 0 & 0 & 0 & 2 \end{bmatrix} \end{array} \begin{matrix} 1 \\ 2 \\ 3 \\ R \end{matrix} , \quad D = \begin{array}{c} \begin{matrix} 1 & 2 & 3 & R \end{matrix} \\ \begin{bmatrix} 0 & -0.5 & 0.5 & 1 \end{bmatrix} \end{array} \; ADP$$

Component	Marginal net benefits	Density factors
G	$\eta_G = \frac{1}{P}\left(\frac{p_2}{k_2}\frac{\partial k_2}{\partial b_G} + \frac{p_3}{k_3}\frac{\partial k_3}{\partial b_G} - \frac{\mu}{k_1}\right)$	$\kappa_G = 1$
ATP	$\eta_{ATP} = \frac{1}{P}\left(\frac{p_3}{k_3}\frac{\partial k_3}{\partial b_{ATP}} + \frac{p_R}{k_R}\frac{\partial k_R}{\partial b_{ATP}} - \frac{0.5\mu}{k_1} - \frac{\mu}{k_2}\right) - 0.5\eta^c_{ADP}$	$\kappa_{ATP} = 0.5$
AA	$\eta_{AA} = \frac{1}{P}\left(\frac{p_R}{k_R}\frac{\partial k_R}{\partial b_{AA}} - \frac{1.5\mu}{k_1} - \frac{\mu}{k_2} - \frac{2\mu}{k_3}\right) + 0.5\eta^c_{ADP}$	$\kappa_{AA} = 1.5$
P	$\eta_P = \frac{1}{P}\left(1 - \frac{2\mu}{k_1} - \frac{2\mu}{k_2} - \frac{2\mu}{k_3} - \frac{2\mu}{k_R}\right) + \eta^c_{ADP}$	$\kappa_P = 2$
ADP	$\eta^c_{ADP} = \frac{1}{P}\frac{p_2}{k_2}\frac{\partial k_2}{\partial b_{ADP}}$	

Supplementary Figure 1. Examples of balanced growth models and their mathematical description. These are derived from the active matrix A and the kinetic functions $k_j(\mathbf{a})$: basis matrix B, investment matrix B^{-1}, closure matrix C, dependence matrix $D = CB^{-1}$, marginal net benefits η_i, and density factors κ_i. (**a**) A model with a simple linear network of irreversible reactions, connecting a single transporter to the final production of proteins[12,13]; linear networks never have dependent reactants, as the number of reactions equals the number of components ($n = m+1$). (**b**) A more elaborate, nonlinear model of irreversible reactions that includes cofactors and a dependent reactant (ADP). Basis components (green) have associated density factors, while the dependent reactants ADP (gray) have no associated density factors.

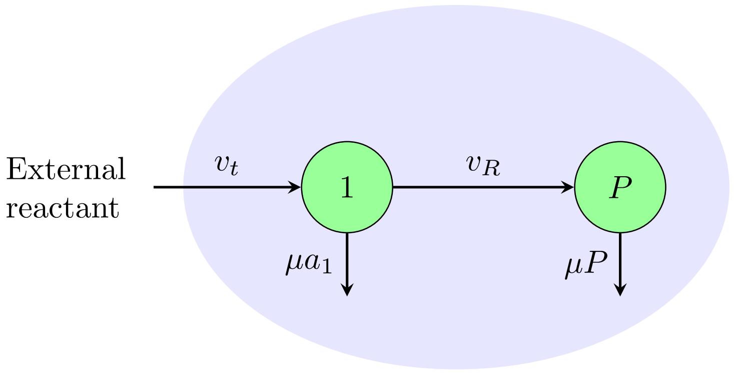

Supplementary Figure 2. A minimal whole-cell model. The model comprises a transport reaction (with rate v_t) and the ribosome reaction (with rate v_R). The arrows labeled μa and μP indicate the dilution of a and P, respectively, at volume growth rate μ.

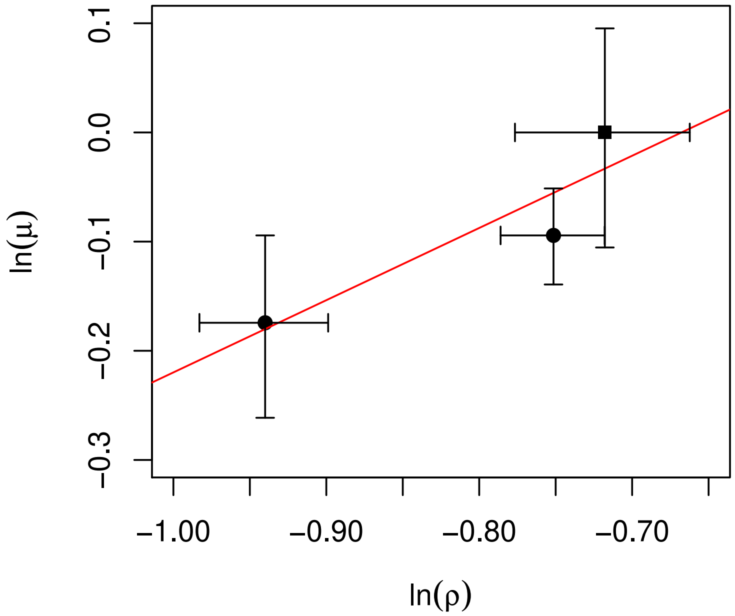

Supplementary Figure 3. Dependence of the growth rate ($\ln \mu$) **on dry weight per free water volume** ($\ln \rho$) **in *E. coli* grown at different external osmolarities**[14]. The square (■) indicates the normal environmental conditions, which correspond to the maximal growth rate; dots (●) indicate growth at lower osmolarities. The red line shows the predicted slope = 0.66, drawn through the center of gravity of the three data points (the line is indistinguishable from the linear regression, also with slope 0.66). Error bars are based on the reported experimental s.d..

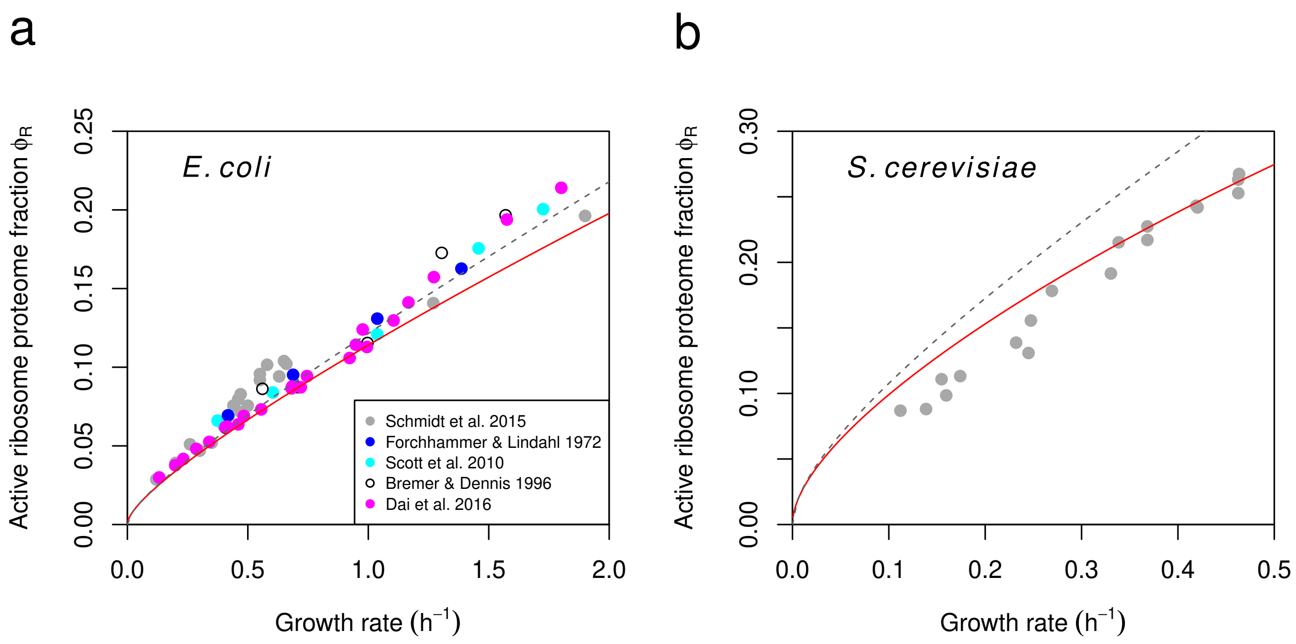

Supplementary Figure 4. An approximation ignoring metabolite dilution. An approximation (dashed grey line, equation 36) that ignores the dilution of intermediates and hence production costs results in good predictions of experimentally observed active ribosome proteome fraction[15,16] at low to intermediate growth rates in *E. coli* (**a**; see also Ref.[17]), but overestimates ϕ_R in yeast (**b**). For comparison, we also show the full GBA predictions (red line, identical to Fig. 2).

Supplementary Tables

Supplementary Table 1. Experimental data from Cayley *et al.*[14]. The table lists cellular free water content $\overline{V}_{free}$ and growth rate μ across different external osmolarities, together with the respective cellular dry weight per cellular free water volume, calculated as $\rho = 1/\overline{V}_{free}$.

Osmolarity (Osm)	$\overline{V}_{free}$ (ml gCDW^{-1})	ρ (gCDW ml^{-1})	μ (h^{-1})
0.03	2.56 ± 0.10	0.39 ± 0.02	0.84 ± 0.07
0.10	2.12 ± 0.08	0.47 ± 0.02	0.91 ± 0.04
0.28	2.05 ± 0.11	0.49 ± 0.03	1.00 ± 0.10

Supplementary Table 2. Definitions of symbols. For simplicity of notation, we also use P as an index for total protein, and ρ as an index for cellular dry weight per volume.

Symbol	Definition [units]
A	active matrix [mass fraction]
B	basis matrix [mass fraction]
C	closure matrix [mass fraction]
D	dependence matrix
A^{-1}	investment matrix
μ	growth rate $[\text{time}]^{-1}$
P	total protein concentration $[\text{mass}][\text{volume}]^{-1}$
α	reactant index
β	basis reactant index
γ	dependent reactant index
a_α	reactant concentration $[\text{mass}][\text{volume}]^{-1}$
b_β	basis reactant concentration $[\text{mass}][\text{volume}]^{-1}$
c_γ	dependent reactant concentration $[\text{mass}][\text{volume}]^{-1}$
j	reaction index
v_j	reaction j flux $[\text{mass}][\text{volume}]^{-1}[\text{time}]^{-1}$
p_j	concentration of protein catalyzing reaction j $[\text{mass}][\text{volume}]^{-1}$
ϕ_j	proteome fraction catalyzing reaction j [mass fraction]
i	protein or independent reactant index ($\in \{P, \beta\}$)
$\mathbf{y}$	vector of concentrations $[\text{mass}][\text{volume}]^{-1}$
$\mathbf{x}$	vector of independent concentrations $[\text{mass}][\text{volume}]^{-1}$
k	kinetic function $[\text{time}]^{-1}$
k_{cat}	turnover number $[\text{time}]^{-1}$
K_{m}	Michaelis constant $[\text{mass}][\text{volume}]^{-1}$
ρ	cellular dry weight per volume $[\text{mass}][\text{volume}]^{-1}$
f	fitness
η_i^0	direct marginal net benefit of i $[\text{volume}][\text{mass}]^{-1}$
η_i	marginal net benefit of i $[\text{volume}][\text{mass}]^{-1}$
η_ρ	marginal benefit of the cellular density $[\text{volume}][\text{mass}]^{-1}$
η_γ^{c}	marginal net benefit of γ $[\text{volume}][\text{mass}]^{-1}$
q_i^j	marginal production cost of i through reaction j $[\text{volume}][\text{mass}]^{-1}$
u_β^j	marginal kinetic benefit of β through reaction j $[\text{volume}][\text{mass}]^{-1}$
u_γ^j	marginal kinetic benefit of γ through reaction j $[\text{volume}][\text{mass}]^{-1}$
κ	density factor
$\mathscr{L}$	Lagrangian $[\text{time}]^{-1}$
λ_ρ	Lagrange multiplier of the density constraint $[\text{volume}][\text{mass}]^{-1}[\text{time}]^{-1}$
λ_γ	Lagrange multiplier of the dependent concentration γ $[\text{volume}][\text{mass}]^{-1}[\text{time}]^{-1}$

Supplementary References

1. Fisher, R. A. & Bennett, J. H. *The genetical theory of natural selection: a complete variorum edition* (Oxford University Press, 1999).
2. Orr, H. A. Fitness and its role in evolutionary genetics. *Nat. Rev. Genet.* **10**, 531–539 (2009).
3. Chevin, L.-M. On measuring selection in experimental evolution. *Biol. Lett.* **7**, 210–213 (2011).
4. Scott, M., Gunderson, C. W., Mateescu, E. M., Zhang, Z. & Hwa, T. Interdependence of cell growth and gene expression: Origins and consequences. *Science* **330**, 1099–1102 (2010).
5. Hui, S. *et al.* Quantitative proteomic analysis reveals a simple strategy of global resource allocation in bacteria. *Mol. Syst. Biol.* **11**, e784–e784 (2015).
6. Heinrich, R. & Schuster, S. *The Regulation of Cellular Systems* (Springer, 2011).
7. Liebermeister, W. Optimal metabolic states in cells. preprint at: https://www.biorxiv.org/content/10.1101/483867v1. (2018).
8. Liebermeister, W. The value structure of metabolic states. preprint at: https://www.biorxiv.org/content/10.1101/483891v1. (2018).
9. Kleijn, I. T., Krah, L. H. J. & Hermsen, R. Noise propagation in an integrated model of bacterial gene expression and growth. *PLOS Comput. Biol.* **14**, 1–18 (2018).
10. Lee, B. Calculation of volume fluctuation for globular protein models. *Proc. Natl. Acad. Sci.* **80**, 622–626 (1983).
11. Bar-Even, A., Noor, E., Flamholz, A., Buescher, J. M. & Milo, R. Hydrophobicity and charge shape cellular metabolite concentrations. *PLOS Comput. Biol.* **7**, e1002166 (2011).
12. Weiße, A. Y., Oyarzún, D. A., Danos, V. & Swain, P. S. Mechanistic links between cellular trade-offs, gene expression, and growth. *Proc. Natl. Acad. Sci.* **112**, E1038–E1047 (2015).
13. Giordano, N., Mairet, F., Gouzé, J.-L., Geiselmann, J. & de Jong, H. Dynamical Allocation of Cellular Resources as an Optimal Control Problem: Novel Insights into Microbial Growth Strategies. *PLOS Comput. Biol.* **12**, e1004802 (2016).
14. Cayley, D. S., Guttman, H. J. & Record, M. T. Biophysical characterization of changes in amounts and activity of escherichia coli cell and compartment water and turgor pressure in response to osmotic stress. *Biophys. J.* **78**, 1748 – 1764 (2000).
15. Schmidt, A. *et al.* The quantitative and condition-dependent escherichia coli proteome. *Nat. Biotechnol.* **34**, 104–110 (2015).
16. Dai, X. *et al.* Reduction of translating ribosomes enables escherichia coli to maintain elongation rates during slow growth. *Nat. Microbiol.* **2**, 16231 (2016).
17. Dourado, H., Maurino, V. G. & Lercher, M. J. Enzymes and substrates are balanced at minimal combined mass concentration in vivo. preprint at: https://www.biorxiv.org/content/10.1101/128009v1. (2017).
